# Malignant Tumor of the Ovary—Exploratory Incision—Recovery

**Published:** 1872-11

**Authors:** Robert Battey

**Affiliations:** Rome, Georgia


### MALIGNANT TUMOR OF THE OVARY—EXPLORATORY INCISION—RECOVERY.

BY ROBERT BATTEY, M.D., ROME, GEORGIA.

Miss Martha, æt. thirty-eight, of large frame, and formerly robust constitution, consulted me on the 8th of August, for a swelling of the abdomen, which she supposed to be dropsical. She had enjoyed excellent health up to 1864, when she suffered from acute rheumatism, which has troubled her several times since. About the first of April last the abdomen was observed to be enlarging, and this augmentation in bulk has continued with increasing rapidity. There may have been some abdominal increase previously, but not to fix her attention and arouse apprehensions in her mind until April, since which time her general health has given way very decidedly.

There is pain in the abdomen, for the most part of a dull aching character, with occasional keen darts. She complains of difficult micturition, and also of pressure upon the rectum. She says that she has lost flesh decidedly since spring, and observes that the abdomen is enlarging very rapidly within the last few weeks. She has a pinched expression of the countenance, reminding one of the "ovarian cachexia," described by Mr. T. Spencer Wells.

*Examination—Palpation.*—A very broad and firm mass occupies the abdominal cavity, extending from side to side, and from a point just below the ensiform cartilage to the pubis. The right illiac fossa is filled by the tumor, while the left is comparatively free. The growth is imperfectly separable by deep grooves into two large lobes, and a left and a right hand lobe above lying to the right and left of the umbilicus respectively, and a third and smaller lobe occupying the right illiac fossa. The whole upper mass of the tumor is freely movable in the abdomen, and falls from side to side as she turns in bed. The tumor gives to the touch an obscure sense of fluctuation, by no means marked.

*Vaginal Taxis.*—Hymen intact; vagina small and contracted, both in its length and breadth. The cervix uteri is small and virginal. It is displaced forward and pressed firmly against the upper surface of the pubic arch and neck of the bladder. The rigid Simpson's womb sound entered but an inch and a half within the uterus. The cul-de-sac of Douglass and pelvic cavity generally, is occupied by a firm, somewhat irregular mass, which cannot be forced upward by moderate pressure.

The menses are regular, abundant and painful; bowels regular; micturition rather difficult and troublesome.

*Diagnosis.*—Composite tumor of right ovary, with much solid material.

*Prognosis.*—Decidedly unfavorable.

She was advised to return to her home, and present herself again in two weeks for further consideration.

Tuesday, August 20.—Re-examined. Her menses ceased on Saturday last. The abdomen presents a decidedly more prominent *anterior* projection than when previously seen on the 8th. There is now evidence of a broad cyst containing fluid, which seems to have mounted up in front of the solid masses, and which fluctuates very sensibly. It is irregular in its outline; lies around the umbilicus and stretches far around into the right hypochondriac and illiac regions, and also into the left illiac, where there is no solid mass, but is entirely absent in the left hypochondrium.

This cyst, if cyst it be, has evidently mounted up over the solid tumors since I saw her last. She is conscious of the fact that the abdomen has sensibly enlarged, and this altogether anteriorly, in the twelve days since her last visit. The enlargement is not in the solid material, which is now less prominent than before. There is resonance on percussion in the gastric left hypochondriac spaces; every where else is dull.

*Per Vaginam.*—The pelvis is blocked up by solid material, which presents a lobulated arrangement and dips down deeply into Douglass' space toward the coccyx. There is no where any fluctuation. The uterus is forced upward and forward above the pubis, the cervix impinging strongly upon the neck of the bladder. The cervix uteri is small. The uterine cavity received the sound to the depth of two and a half inches.

There seems to be an *ill-defined* movement of the uterus separately from the mass which surrounds it; but this movement is limited by the general blocking up of the pelvis by the tumor, which can be elevated somewhat, but cannot be lifted out of the pelvic basin by any justifiable force.

*Per Rectum.*—The tumor is felt occupying the recto-vaginal pouch, chiefly upon the left side, forcing itself low down toward the coccyx and displacing the rectum toward the right side. There is no fluctuation anywhere.

*Diagnosis.*—Composite tumor of the right ovary, with much solid material, free of abdominal adhesions. Probably large cyst expanded over the front of the tumor. Probably pelvic adhesions. Suspect malignancy of the tumor.

*Prognosis.*—Very unfavorable.

*Remarks.*—She was advised that this tumor must certainly destroy life at no distant period if allowed to continue its course; that the prospects of a successful ovariotomy were not flattering—were indeed gloomy. She was further advised that the rate of increase of the abdominal distension indicated an early necessity for some means of evacuating the fluid; that an exploratory incision would do this with but little greater risk than tapping, with the additional advantages of diagnosis of the precise character and relation of the tumor, and an immediate ovariotomy, if it should be found practicable and expedient.

The patient, a lady of uncommon nerve and force of character, responded at once and decisively: "Doctor, this tumor must come out if it is possible."

September 3.—Re-examined by myself, in conjunction with my friends, Drs. G. W. Holmes and W. D. Hoyt, at the Rome Hotel. There is a markedly increased enlargement since I last examined her on the 20th August; and this increased development is altogether forward, not laterally. There is now a good deal of fluctuation in front of the solid masses. The umbilicus is protuberant for the first time. She complains considerably of pain in the left hypochondrium. There is perceptible change in the position of the solid masses in the abdomen. It is agreed that there are no adhesions of the tumor in the abdomen; that the tumor is probably but not positively ovarian in its origin; that the fluid is probably cystic and not ascites.

*Per Vaginam*—by myself. The mass in the pelvis seems to have risen some what toward the abdomen. The pelvis is not so closely jammed by it, nor does it extend so low down in

Douglass' space. There is less pressure upon the rectum and less difficulty in micturition. The uterus is higher up, and has lost its central position—is pushed rather over toward the left side. The patient being upon her back, I am now able to push up the mass a perceptible distance toward the abdomen by my finger. She feels distinctly that it *does* move upward as I make the pressure.

She is sensibly weaker than on the 20th August, and gets about with increasing difficulty. She was not able to ride to Rome in a carriage to-day, and could not walk from the depot as heretofore.

Saturday, Sept. 14.—Visited her at her home in the country. She suffered considerable pain for some days following her last visit to Rome. She is just finishing her menstrual period. For three days past she has been more comfortable than usual. Since her last visit to Rome the pain has been altogether in the epigastrium and right hypochondrium. All pelvic pain is gone. She has now no trouble with the bladder, and the bowels are quite regular and require no laxatives. In a word, she "feels that the pressure down below is relieved." The tongue is clean, appetite fair, digestion good.

I find the abdomen more enlarged than at the last examination, but the enlargement is now lateral as well as anterior. In the supine posture the forward projection is not so prominently marked. There is still the same fluctuation in front as of a cyst overlying the solid mass; but there is likewise well marked fluctuation in the flanks, with dull percussion on both sides. There seems to be a "water line" on each side, and this line changes with change of posture. The solid mass moves very freely in the abdomen—much more freely than at any previous examination.

*Per Vaginam.*—I find the pelvic mass has risen decidedly toward the abdomen. It is much more movable and recedes upward before the finger more markedly than heretofore. The pelvic portion of the tumor moves synchronously with the abdominal, showing an intimate connection between the two. The uterus also appears to move with the tumor; but this is not very definitely made out. The uterus is still jammed by the tumor closely against the pelvic ramus of the left side, but is not fastened there so firmly as heretofore.

*Diagnosis.*—Composite tumor of right ovary with ascites, probably anterior cyst, possibly malignant.

*Prognosis.*—More favorable as regards adhesions; otherwise not promising.

*Remarks.*—The patient is still very resolute in her determination to "have something done." She is much more hopeful of the issue than I am; counts much upon the force of her resolution and her former strong constitution. She will have castor oil to-morrow night; rest Monday; come to Rome on Tuesday for exploratory operation on Wednesday. It is understood that a small incision will be made, the fluids removed, the tumor examined, and if its character and relations will admit of it, an immediate extirpation will be effected; otherwise the incision will be closed and the tumor allowed to remain undisturbed.

Tuesday, September 17.—Came up by rail to-day. I saw her at 1 P. M., in bed; pulse 100. This trip has worried her considerably. She complains of soreness and pain. The oil on Sunday night purged actively and required checking. Her tongue is clean; appetite fair; mind cheerful and hopeful. Ordered a quarter grain morphia and rest.

Six P. M.—Pulse 88; strong; no heat of skin; no headache. Ordered morphia for the night and enema for the early morning.

*First day.*—Wednesday, September 18, 9 A.M.—Pulse 96; copious enema returns without fæces. She is somewhat excited by the near approach of the operation. Ordered brandy and rest.

Twelve M.—Put her upon the table; had quarter grain morphia. Drs. G. W. Holmes, W. D. Hoyt, J. M. Gregory, W. L. Selman and J. B. S. Holmes present.

Dr. Hoyt began the administration of sulphuric ether at 12:15 P.M. A space of 30 minutes was required for full anæsthesia, when the incision was made through the abdominal wall in the central line beginning an inch and a half above the pubis and extending upward three inches to admit of the introduction of the hand for thorough exploration. On opening the peritoneum the end of an elastic tube was introduced and the lips of the wound held around it in such a manner as to draw off about twenty-two pounds of ascitic fluid into the receiving vessel. Probably three pounds additional of this fluid was lost upon the clothes arranged for protection. The fingers and hand were now passed into the cavity and dipped down into the pelvis, as well as around the tumor in every direction, as far as could be conveniently reached. There was no anterior cyst. The tumor appeared to be solid. The abdominal portion, constituting the great bulk of the mass was entirely loose and freely movable. The pelvic portion was slightly adherent to the uterus and sprang by a pedicle from its right wing. No very careful search for adhesions was instituted, however, for the consistency and texture of the tumor, as brought into view, coupled with its general nodular character, stamped it as malignant in its type, and this diagnosis of malignancy was further confirmed by the presence of very numerous little nodular masses studding the parietal peritoneum, varying in size from small shot to that of marbles.

There was reason for the belief that the tumor could be lifted out and removed with some degree of facility, and there certainly was strong temptation to go on in that direction. But as the disease had already asserted itself in the walls of the abdomen, as there might be adhesions of a more formidable kind behind and below the tumor, as it would be necessary to extend the incision greatly for the removal of a solid mass of certainly twenty and possibly of thirty pounds weight, it was deemed inadvisable to add so much to the hazard already incurred, when nothing but a temporary palliation could be promised in return. The wound was, therefore, closed by five deep sutures and other superficial sutures, all of silver wire, and the patient was put to bed at 1:40 P.M. There was vomiting several times while under the influence of the ether. She rallied promptly with a good pulse and complained of no pain. Had a little brandy and a half grain morphia. 3 P.M.—Pulse 112; resting well; dozes a little. She is greatly disappointed in her hope that the tumor would be removed. 6 P.M.—Pulse 100; is quiet; has some nausea; no vomiting; a little abdominal pain. 8 P.M.—Pulse 108; strong and full. 9½ P.M.—Pulse 120; resting well; no pain; drew 8 ounces normal urine; she had a half grain morphia during the night.

*Second day.*—6 A.M.—Pulse 108; a little thrill; resting quietly; drew 8 ounces normal urine. 12 M.—Pulse 108; rests well; no pain; no morphia; a rash on the arms. 1 P.M.—Had a quarte[illegible]

grain morphia. 2 P.M.—Pulse 96; wound looks well; drew 5 ounces healthy urine. 7 P.M.—Pulse 100; resting quietly. 9 P.M.—Pulse 108; complains of sour stomach from a little buttermilk at dinner; drew 4 ounces urine; gave lime water and half grain morphia; she has brandy four or five times daily. 10½ P.M.—Quiet but not sleeping.

*Third day.*—7 A.M.—Pulse 108; a little nausea; gentle perspiration; drew 4 ounces urine. 9 A.M.—Pulse 100; has vomited a sour, greenish liquid, with sense of relief; to have lime water and elm mucilage. 12½ P.M.—Pulse 108; nausea continues; no opiate since last night. 2½ P.M.—Pulse 108; still some nausea; drew 5 ounces urine; had quarter grain morphia. 4 P.M.—Bilious vomiting. 6 P.M.—Pulse 108; still nauseated. 9 P.M.—Pulse 104; skin natural; resting quietly; a little nausea; drew 6 ounces urine.

*Fifth day.*—7 A.M.—Pulse 96; the bowels were moved by enema last night; urine passed voluntarily; slept well; there is still some nausea and vomiting; the lips of the wound are firmly adherent and look healthy; removed a superficial suture. 5½ P.M.—Pulse 100; quiet and resting; nausea allayed by creasote mixture; complains of uneasiness in the bowels. 10 P.M.—Pulse 92; has slept some; bowels moved by enema, with expulsion of considerable flatus; urine passes naturally; vomited once; nausea less troublesome; takes lime water and milk; had quarter grain morphia.

*Sixth day.*—7 A.M.—Pulse 92; rested well; urine normal; nausea gone; continues the creasote. 1 P.M.—Pulse 96; no nausea; bowels moved by enema; had quarter grain morphia per anum; continues creasote. 6 P.M.—Pulse 92; skin moist; has been sleeping quietly; "feels like a new creature;" take gruel and milk. 9½ P.M.—Pulse 92; quiet and comfortable; in a joking humor.

*Seventh day.*—4 A.M.—Had flatulence and much pain in the bowels; a quarter grain morphia did not relieve it; warm water enema expelled flatus with relief; had another quarter grain morphia per anum. 7½ A.M.—Pulse 92; resting easy; no pain; no nausea; wound healthy. 1 P.M.—Pulse 84; resting quietly; takes creasote; milk and lime water. 6 P.M.—Another expulsion of flatus by enema; sleeping quietly. 9 P.M.—Pulse 96; complains a little of abdominal pain; had no morphia to-day. 10 P.M.—Had quarter grain morphia.

*Eighth day.*—11 A.M.—Pulse 100; had a quiet, comfortable night; no nausea and very little pain. 6 P.M.—Passed the day comfortably; bowels moved by enema; had quarter grain morphia. 9 P.M.—Pulse 100; some pain; had a quarter grain morphia.

*Ninth day.*—3 P.M.—Pulse 108; nausea and vomiting this morning; no morphia since last night; rests well now; some uterine bleeding to-day. 9 P.M.—Pulse 100; bilious vomiting; spontaneous movement of the bowels; had a fractional dose of seidlitz; to be repeated every four hours.

*Tenth day.*—8 A.M.—Pulse 104; "had a sick night;" still much nausea; ordered shaved ice; fractional seidlitz every two hours; camphor water; oxalate cerium. 9 P.M.—Pulse 112; nausea gone; tongue red and pointed; cerium has stayed the stomach; bowels moved naturally; she had no morphia since last night; no pain; soreness of the abdomen subsiding.

*Eleventh day.*—8 A.M.—Pulse 92; had a good night; some nausea, and vomited once; she takes egg-nog, which agrees well; removed all of the deep sutures; there is some pus in the track of each wire; wound is evenly united throughout. 2 P.M.—Pulse 100; easy and quiet; complains only of a sense of heat. 9 P.M. Pulse 100; comfortable; no nausea; no pain; requires no anodyne.

*Twelfth day.*—9 A.M.—Pulse 100; rested well last night. 9 P.M. Pulse 100; very comfortable day.

*Thirteenth day.*—9 A.M.—Pulse 104; good night; appetite better; takes diet; bowels moved by enema. 8 P.M.—Pulse 92; comfortable day.

*Fourteenth day.*—10 A.M.—Pulse 92; oysters for breakfast, and egg-nog. 6 P.M.—Pulse 88; enjoyed an oyster dinner.

*Fifteenth day.*—Pulse, A.M., 88—P.M., 80; comfortable; appetite improving; takes oysters, buttermilk, and toast.

*Sixteenth day.*—Pulse, A.M., 80—P.M., 84; sat up a little in an arm-chair.

*Seventeenth day.*—Pulse, A.M., 96—P.M., 96; not so comfortable as yesterday; had seidlitz, and an emulsion of turpentine.

*Eighteenth day.*—Pulse, A.M., 80—P.M., 96; don't feel so well, but has no pain; no nausea; mouth rather dry; continue turpentine.

*Nineteenth day.*—Pulse 100; sat up an hour in a chair; tongue clean, but too red.

*Twentieth day.*—Pulse, A.M., 100—P.M., 88; has broiled chicken for dinner.

*Twenty-first day*—Pulse, A.M., 88—P.M., 88; bowels purged freely by seidlitz.

*Twenty-second day.*—Very comfortable every way.

*Twenty-third day.*—Went home in an arm-chair, by rail.

*Twenty-eighth day.*—Visited her at her home in the country; found her very comfortable and cheerful. She suffers little or no pain; says she has experienced a marked relief from the pain which she suffered for months prior to the operation. The wound is smoothly cicatrized throughout. There is a small nodular tumor at the navel, about the size of a small grape, with an expanded vein running across its surface. It is similar in character to those found upon the peritoneal aspect of the abdominal wall. The abdomen is not larger now than it was immediately after the operation. I can detect no fluid anywhere. Her appetite is fair, digestion good, bowels regular; does not require purgatives. The micturition and defecation are unimpeded. She sleeps well; does not need anodynes, nor indeed any medicine. In answer to my question—"Miss M——, in view of your experience, if this operation were to be done over again, under similar circumstances, what would be your determination?"—she promptly responded: "Why, Doctor, I would do exactly as I did do, and say exactly what I did say. I am every way more comfortable; my mind is at rest; I feel that I have discharged a duty in availing myself of the only chance for cure."

*Fortieth day.*—Complains of more pain, but otherwise is as before; she is able to move about the house a little; there is no evidence of any increased abdominal enlargement; the tumor is now very generally adherent to the abdominal parietes, and hence is much less movable.

*Forty-third day.*—Was called to her mother. Miss M—— is suffering but little pain, and reports herself to be gaining strength.

